# Thrombosis and risk factors: A comment

**DOI:** 10.5505/tjh.2012.83713

**Published:** 2012-03-05

**Authors:** Selami Koçak Toprak, Yunus Kasım Terzi, Feride Şahin

**Affiliations:** 1 Baskent University, Department of Hematology, Ankara, Turkey; 2 Baskent University, Department of Medical Genetics, Ankara, Turkey

## TO THE EDITOR

We read with great interest the recent publication byAkar related to thrombosis and risk factors, in which hereached in conclusion that in case of need only homocysteine(Hcy) levels should be routinely analyzed and notthe 5, 10-methylenetetrahydrofolate reductase (MTHFR)677 T polymorphism [1]. The methylation of Hcy tomethionine is catalyzed by the MTHFR enzyme. As far asis known, genetic deficiency of MTHFR is one cause leadingto increased plasma Hcy levels [2]. But, it should notbe forgotten that Hcy rises in many acquired and geneticconditions (Table) [3]. There are three main indications fordetermining plasma total Hcy: (a) to diagnose homocystinuria;(b) to identify individuals with or at risk of developingcobalamin or folate deficiency; (c) to assess total Hcyas a risk factor for cardiovascular and other disorders [4]. There is an involved question in this point: Is increasedplasma total Hcy level related to both venous and arterialocclusive disease? And, if is it true, are polymorphism andmutation of MTHFR directly playing a role to augment thelevel of Hcy for cardiovascular disease (CVD)? As reviewedelsewhere, moderately increased plasma Hcy is associatedwith venous and arterial occlusion [5]. Moreover, as it isknown, the presence of MTHFR 677C→T polymorphismis a strong risk factor for increased plasma Hcy level butnot for CVD [4]. In a metaanalysis including 11.162 CVDcases and 12.758 controls, with high Hcy levels in a stateof low folate levels, the TT genotype was associated witha 16% increase in coronary heart disease risk [6]. Concordantly,previous studies had shown that the MTHFR 677C→T polymorphism is only associated with high Hcylevels or increased CVD risk in a setting of low folate status [6]. Hence, at higher dietary intakes of folate, the effectof the MTHFR 677C→T genotype has no adverse effecton plasma Hcy levels or on subsequent risk of CVD. Theresults support the hypothesis that impaired folate metabolism,resulting in high Hcy concentrations, plays a causalrole in the occurrence of CVD. In view of cost effectiveness,do not investigate routinely MTHFR 677C→T polymorphismin the general or CVD population seems to bereasonable, but the other mutations of MTHFR could bestill influential for high plasma Hcy levels.

In conclusion, even if some researchers contradict, providedthat folate status is adequate, there is little clinicalvalue of screening for MTHFR 677C→T genotype in thegeneral population for prediction of venous and arterialocclusive disorders and high Hcy levels of course.

## CONFLICT OF INTEREST STATEMENT

The authors of this paper have no conflicts of interest,including specific financial interests, relationships, and/or affiliations relevant to the subject matter or materialsincluded.

## Figures and Tables

**Table 1:**
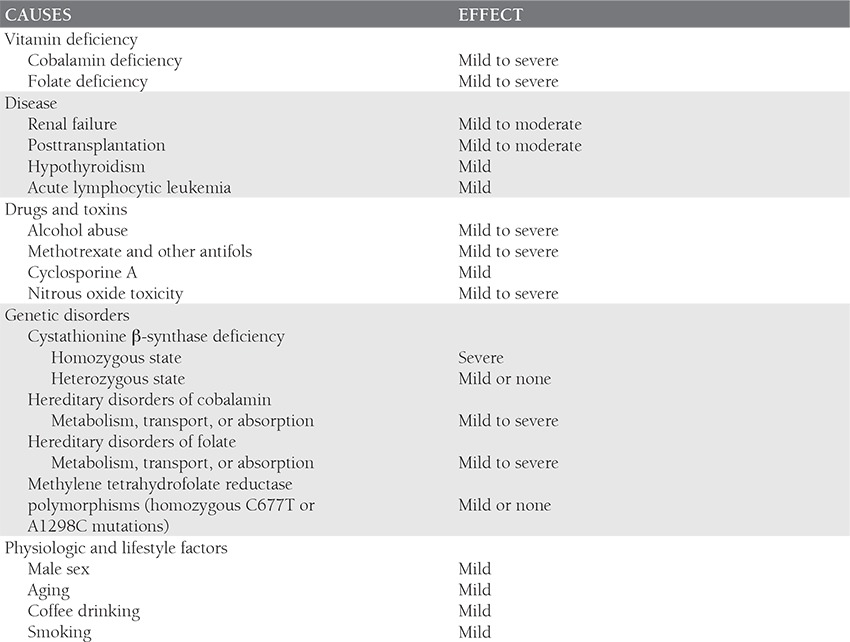
Causes of Elevated Homocysteine Levels
